# The notch target gene *HEYL* modulates metastasis forming capacity of colorectal cancer patient-derived spheroid cells in vivo

**DOI:** 10.1186/s12885-019-6396-4

**Published:** 2019-12-03

**Authors:** Sarah Weber, Sebastian E. Koschade, Christopher M. Hoffmann, Taronish D. Dubash, Klara M. Giessler, Sebastian M. Dieter, Friederike Herbst, Hanno Glimm, Claudia R. Ball

**Affiliations:** 1 Translational Functional Cancer Genomics, National Center for Tumor Diseases (NCT) and German Cancer Research Center (DKFZ), Heidelberg, Germany; 2 German Cancer Consortium (DKTK) Frankfurt am Main, Frankfurt am Main, Germany; 3 Department of Hematology and Oncology, University Hospital Frankfurt, Frankfurt am Main, Germany; 4 Department of Translational Medical Oncology, National Center for Tumor Diseases (NCT) Dresden and German Cancer Research Center (DKFZ), Dresden, Germany; 5 Center for Personalized Oncology, University Hospital Carl Gustav Carus Dresden at TU Dresden, Dresden, Germany; 6 German Cancer Consortium (DKTK) Dresden, Dresden, Germany

**Keywords:** Colorectal cancer, Metastasis, Xenotransplantation, HEYL

## Abstract

**Background:**

While colorectal cancer (CRC) patients with localized disease have a favorable prognosis, the five-year-survival rate in patients with distant spread is still below 15%. Hence, a detailed understanding of the mechanisms regulating metastasis formation is essential to develop therapeutic strategies targeting metastasized CRC. The notch pathway has been shown to be involved in the metastatic spread of various tumor entities; however, the impact of its target gene *HEYL* remains unclear so far.

**Methods:**

In this study, we functionally assessed the association between high HEYL expression and metastasis formation in human CRC. Therefore, we lentivirally overexpressed HEYL in two human patient-derived CRC cultures differing in their spontaneous metastasizing capacity and analyzed metastasis formation as well as tumor cell dissemination into the bone marrow after xenotransplantation into NOD.Cg-Prkdc^scid^ Il2rg^tm1Wjl^/SzJ (NSG) mice.

**Results:**

HEYL overexpression decreased tumor cell dissemination and the absolute numbers of formed metastases in a sub-renal capsular spontaneous metastasis formation model, addressing all steps of the metastatic cascade. In contrast, metastatic capacity was not decreased following intrasplenic xenotransplantation where the cells are placed directly into the blood circulation.

**Conclusion:**

These results suggest that HEYL negatively regulates metastasis formation in vivo presumably by inhibiting intravasation of metastasis-initiating cells.

## Background

Metastasis and its consequences are the main causes of cancer-related mortality [1, 2]. About 25% of colorectal cancer (CRC) patients already present synchronous metastases at initial diagnosis, and nearly 50% develop metachronous metastases [3]. The five-year survival rate for CRC patients with localized disease amounts to 90.1%, whereas the rate drops to 13.5% in patients with distant spread [4]. More than half of the deaths from CRC have been ascribed to the presence and progression of liver metastases [5].

Metastasis formation is driven by a multistep invasion-metastasis cascade [6, 7]. One pathway involved in the metastatic spread is the notch pathway, which enhances the initiation of epithelial-mesenchymal transition (EMT), promotes anoikis resistance, causes the production of dysfunctional hyperpermeable tumor vessels and supports the formation as well as the maintenance of tumor stem cells [8, 9]. *HEYL*, *HEY1* and *HEY2* are notch target genes belonging to the hairy and enhancer of split-related (HESR)-family [10–12]. These HEY factors function as repressive transcription factors by forming homo- or heterodimers and interact directly with the transcription machinery or local chromatin at active promoter regions [12, 13]. Thereby, they seem to function in highly redundant ways due to sequence similarities and overlapping target genes [12, 14]. During embryonic development, they are involved in somatogenesis, neurogenesis and vascular as well as cardiogenic development [15–17]. While *HeyL* knock-out mice lack a phenotype, a simultaneous knock-out of *HeyL* and *Hey1* in mice causes severe cardiac malformations with membranous ventricular septal defects and dysplastic valves due to an impaired EMT of endocardial cells [18]. A correlation between notch pathway activation, HEY factor expression and the activation of EMT markers was also found in breast cancer [19, 20]. Furthermore, accumulating evidence indicates that HEY factors exert both tumor-enhancing [19–23] and tumor-inhibiting [24, 25] effects depending on the respective tumor entity. In human CRC, endogenous overexpression of HEY1 was described as a negative prognostic factor that correlates with perineural as well as vascular invasion, lymph node metastases and inversely with microsatellite instability (MSI) [26].

Based on these first hints we hypothesized that HEY factors may play a role in the metastatic process of CRC. To address this question, we overexpressed HEYL in patient-derived colorectal cancer cells and assessed the impact of HEYL overexpression on metastasis formation utilizing xenotransplantation models in NSG mice which allows addressing different steps of the metastatic cascade.

## Methods

### Generation and cultivation of cells

Colorectal cancer derived spheroid cultures were generated from tumor tissue of two CRC patients as described earlier [27]. In brief, 2008 and 2012 primary human CRC tissue or derived metastases were obtained at the University Hospital Heidelberg in accordance with the Declaration of Helsinki. Both patients signed informed consent as approved by the Ethics Review Board of the Medical Faculty, University of Heidelberg (approval number 323–2004). The tumor samples were minced and enzymatically digested with dispase (Becton, Dickinson and Company) and DNAse I (Roche). If necessary, mucus was dissolved by sputolysin (Calbiochem). The isolated cancer cells were cultivated in ultra-low attachment flasks (Corning) under serum-free conditions with addition of 10 ng/ml fibroblast growth factor (R&D Systems) and 20 ng/ml epidermal growth factor (R&D Systems) as described previously [27]. Under these conditions, multicellular spheroids formed. These spheroid cultures were authenticated and checked for contaminations with Multiplexion [28]. HEK-293 T cells were cultured under the same conditions as the patient-derived CRC cells. The morphology of the cells was examined via microscope (Fluorescence microscope Axiovert 200, Zeiss).

### Lentiviral vector production and transduction

Human *HEYL* coding sequence (Entrez Gene ID: 26508, transcript HEYL-201) was cloned into pRRL_CMV_*eGFP*_WPRE, a third generation self-inactivating lentiviral vector based on the human immunodeficiency virus type 1 (HIV-1) encoding for a *HEYL*-IRES-*eGFP* sequence under the control of a human CMV promotor. An empty vector encoding only for *enhanced green fluorescent protein* (*eGFP)* under the control of a human CMV promotor (HEK-293 T and M1: CMV-*eGFP*, NM1: CMV-IRES-*eGFP*) was used as a control for the overexpression experiments. Vector particle-containing supernatant was generated as described by Dull et al. with polyethylenimine (Sigma) for transfection and concentrated via ultracentrifugation [29]. Dissociated cells were transduced with Viromag R/L beads (OZbiosciences) at multiplicities of infection (MOI) ranging from 1 to 40 determined by virus titration in HeLa cells and subsequent flow cytometry analysis. GFP positive transduced cells were selected by fluorescent associated cell sorting (FACS).

### Xenotransplantation models

In vivo xenotransplantations were performed with male or female NOD.Cg-Prkdc^scid^ Il2rg^tm1Wjl^/SzJ (NSG) mice. They were purchased from The Jackson Laboratory and further expanded in the Centralized Laboratory Animal Facilities of the German Cancer Research Center of Heidelberg. All mice were housed in a strict pathogen-free (SPF) animal facility according to the recommendations of the the Federation for Laboratory Animal Science Associations (FELASA). Animals were group-housed in standard individually ventilated cages with ad libitum diet (autoclaved mouse/rat housing diet 3437; Provimi Kliba AG), autoclaved tap water, wood chip embedding (LTE E-001, ABEDD) and nesting material. The light/dark cycle was adjusted to 14:10 with lights on at 6:00 am and light off at 8:00 p.m. Room temperature and relative humidity were adjusted to 22.0 ± 2.0 °C and 55.0 ± 10.0% respectively. All animal experiments performed in this study were conducted according to the national guidelines and were reviewed and confirmed by an institutional review board/ethics committee headed by the responsible animal welfare officer. The Regional Authority of Karlsruhe, Germany finally approved the study protocol involving xenotransplantation of human derived tissue in immune deficient mice as the responsible national authority (approval numbers G-129/07 and G-228/12).

For xenotransplantation, 1 × 10^5^ HEYL-overexpressing or control cells in 25 μl growth medium were mixed 1:1 with Matrigel (BD Biosciences) and subsequently injected under the kidney capsule of NSG mice (*n* = 6 single animals per group) or into the spleen of NSG mice (n = 6 single animals per group). Prior to surgery, mice received metamizole (200 mg per kg of body weight) as analgesic. Anesthesia was performed with 1.75% isoflurane in the breathing air. Mice were checked at least daily following transplantation and sacrificed immediately by cervical dislocation when reaching the specific ethical human end point criteria of the approved animal experiments. Specifically, human endpoints were defined as occurrence of relevant changes in behaviour or signs of pain (weight loss > 10%; unphysiological body posture; reduced food and water intake; swollen abdomen) or when tumors reached palpable size following intrarenal or intrasplenic transplantation. Two experimental mice died spontaneously during the experiment without a palpable tumor or the appearance of other endpoint criteria (1x M1 sub-renal capsular HEYL and 1x NM1 intrasplenic control)**.** The primary tumor, all organs with visible metastases and both femurs were extracted. Tumor weight defined as the weight of the primary tumor plus the organ of transplantation (kidney or spleen, respectively) was recorded. Time to tumor growth was defined as time from xenotransplantation until sacrifice of the mouse. Metastasis formation capacity was evaluated by monitoring the number of mice with metastasis formation, the sites and number of distant metastases and the tumor cell dissemination into the bone marrow. Distant metastases were defined as macroscopically visible metastases in other organs (brain, lungs, heart, thymus, liver, kidneys, gastrointestinal tract, genitals, urinary bladder, bones, and soft tissue) than the primary transplantation site. For sub-renal capsular xenotransplantation, visible metastases in the spleen were not defined as distant metastases since metastases from the left kidney to the adjacent spleen seemed to occur mostly by continuous tumor spread and not exclusively by hematogenous spread. Liver metastases were counted up to a maximum of 50 metastases. Subsequently, primary tumor and metastases tissue were minced into small pieces and frozen at − 80 °C for RNA isolation. Primary tumor and metastases were tested for human markers and *HEYL* expression, via qRT-PCR. Bone marrow cells were isolated from the femurs by flushing the bones with PBS after cutting off the epiphyses. After incubation with Erythrocyte Lysis Buffer (Stemcell Technologies) for 5 min, cells harvested from the bone marrow were cultivated in the same way as spheroid cultures. Tumor cell dissemination into the bone marrow was defined as an outgrowth of tumor spheroids within 1 month. This method had previously been confirmed to detect tumor cells expressing human markers (as measured by qRT-PCR).

### qRT-PCR

RNA from spheroid cultures or tumor tissue was extracted with the AllPrep DNA/RNA Kit (QIAGEN) according to the manufacturer’s instructions. For one-step qRT-PCR, 2.5 ng RNA per sample was used. One-step qRT-PCR was performed by using the QuantiFast SYBR Green RT-PCR Kit (QIAGEN) with gene-specific primer pairs (Additional file 1). All samples were measured in triplicates and normalized using *GAPDH* as housekeeping gene and the same aliquot of HEK-293 T cells as a calibrator (HEK-293 T expression levels were taken as 1.0).

### Immunoblotting

Cellular protein was extracted using 1 μl of RIPA lysis buffer per 5 × 10^4^ cells (150 mM NaCl, 0,5% Na-desoxycholate, 1% NP-40 buffer, 0.1% SDS, 50 mM Tris pH 7.5, protease inhibitor cocktail (Roche Diagnostics)). The protein concentration was determined by BCA protein assay (Thermo Fisher Scientific). Equal amounts of protein were mixed 3:1 with loading buffer (4x Laemmli buffer (BioRad), 10% β-mercaptoethanol). For immunoblotting, 50 μg of protein was separated in a 13% polyacrylamid gel using a Mini Protean Tetra Cell Electrophoresis Module (BioRad). Then, the proteins were transferred on a PVDF membrane via a Trans Blot® SD Semi-Dry Transfer Cell (BioRad). Blocking was performed with 5% milk powder and 0.45% Tween 20 in PBS. Primary antibodies used for detection were anti-human-α-Tubulin (T5168, Sigma-Aldrich; dilution 1:1000) used as an internal control and anti-human-HEYL (HPA001438, Sigma-Aldrich; dilution 1:100). The protein bands were visualized using a chemiluminescence system. HEK-293 T cells transduced with a HEYL overexpression vector were used as a positive control.

### Statistical analysis

For the statistical analysis of publicly available data sets, clinical data, sample information and corresponding RNA-Seq gene expression data (log2(x + 1) transformed RSEM-normalized gene count) of a cohort of 631 patients with colon adenocarcinoma (COAD) and rectal adenocarcinoma (READ) from The Cancer Genome Atlas (TCGA) were retrieved via UCSC Xena [30]. To test the association between *HEYL* expression levels and patient overall survival (OS), all patients with available gene expression data of the tumor tissue and clinical OS data (371 cases) were analyzed. For discrimination of high and low expression levels, the group was split by the median expression level. For an analysis of the association between expression levels and American Joint Committee on Cancer (AJCC) cancer stage, patients with available gene expression data of the tumor tissue and AJCC stage information (410 cases) were included. OS of the TCGA colorectal adenocarcinoma cohort was analyzed by Kaplan-Meier estimation and differences in OS between the groups were tested using the log-rank test. Numbers of patients with AJCC stage IV colorectal cancer in contrast to AJCC stage I-III colorectal cancer were analyzed using the chi-squared test.

Whole human genome expression DNA microarray data sets of patients with CRC were obtained via National Center for Biotechnology Information (NCBI) Gene Expression Omnibus (GEO) [31]. All expression levels were measured as signal intensity within the Affymetrix Human Genome U133 Plus 2.0 Array. Poorly expressed probe sets were filtered out using Gene Pattern [32] with the following parameter settings: floor = 20, ceiling = 20,000, min_fold_change = 3, min_delta = 100 and num_outliers_to_exclude = 2. Only patients with available expression data of the tumors were included in our analysis. Patients were assigned to groups due to the origin of the material (primary lesion or metastatic lesion), respectively.

For xenotransplantation models, differences in primary tumor weight and tumor growth time between experimental and control groups were tested with the Mann-Whitney U test. The proportion of mice with distant metastasis or tumor cell dissemination into the bone marrow was analyzed via a two-sided chi-squared test. The total number of either liver metastases or liver and lung metastases was modelled by a zero-inflated negative binomial regression analysis.

Unless stated otherwise, all statistical analyses were performed with R version 3.4.2.

Levels of significance were defined as follows: *, *p* = 0.05–0.01; **, *p* = 0.01–0.001; ***, *p* < 0,001.

## Results

### *HEYL* expression is associated with metastasis in colorectal cancer patients

To investigate whether *HEYL* expression is associated with metastasis formation in colorectal cancer patients, we analyzed publicly available datasets for correlation between *HEYL* RNA expression levels, OS and metastatic cancer stage and compared *HEYL* RNA expression in metastatic and primary lesions.

**Fig. 1:**
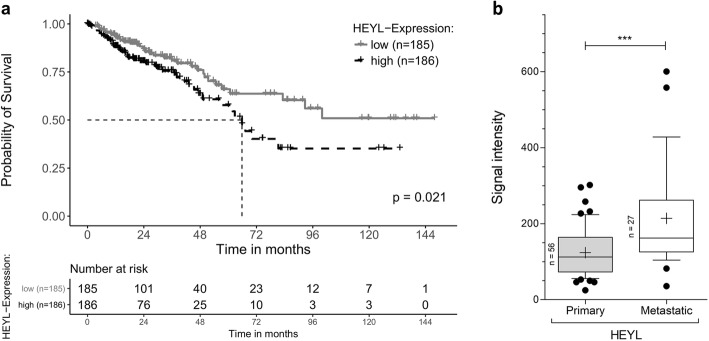
*HEYL* expression in colorectal cancer in the context of metastasis. **a** Kaplan-Meier estimates of overall survival (OS) of colorectal cancer patients within the TCGA stratified by *HEYL* expression within the tumors. High expression is defined as median or higher and low expression as below-median. Median survival is marked by a dashed line. The absolute numbers of patients at risk (n) are depicted below each figure. **b** *HEYL* mRNA expression levels at primary compared to metastatic lesions in a cohort of 83 unresectable colorectal cancer patients prior to chemotherapy [33]. Expression levels were measured as signal intensity within the Affymetrix Human Genome U133 Plus 2.0 Array. Both box plots show mean (+ symbol), median (line), interquartile range (box) range (whiskers) and outsider of the 1.5 interquartile range (dots). The total number of patients per group is depicted on the left side of each box

We first focused on a potential association between OS and *HEYL* expression levels in the TCGA CRC cohorts (TCGA-COAD, TCGA-READ; *n* = 631) and found significantly decreased OS of patients with *HEYL* high-expressing tumors compared to patients with *HEYL* low-expressing tumors (*p* = 0.021) (Fig. 1a). Further, patients with *HEYL* high-expressing tumors showed an increased presence of distant metastasis (AJCC stage IV) with 20.1% (37/184) of the patients classified as stage IV (AJCC classification) compared to 10.6% (24/226) in the *HEYL* low-expressing group (*p* = 0.007). Next, we utilized a gene expression data set of primary tumors or metastases from 83 unresectable CRC patients prior to chemotherapy [33] (GEO accession GDS4393 and GDS4396) to compare *HEYL* expression levels in primary tumors and metastatic lesions and detected a significantly higher expression of *HEYL* in metastatic lesions (*p* = 0.0003) (Fig. 1b). Collectively, these analyses point towards an association between high *HEYL* expression and the presence of metastasis in CRC patients.

### Successful ectopic HEYL overexpression in patient-derived colorectal cancer cells

**Fig. 2:**
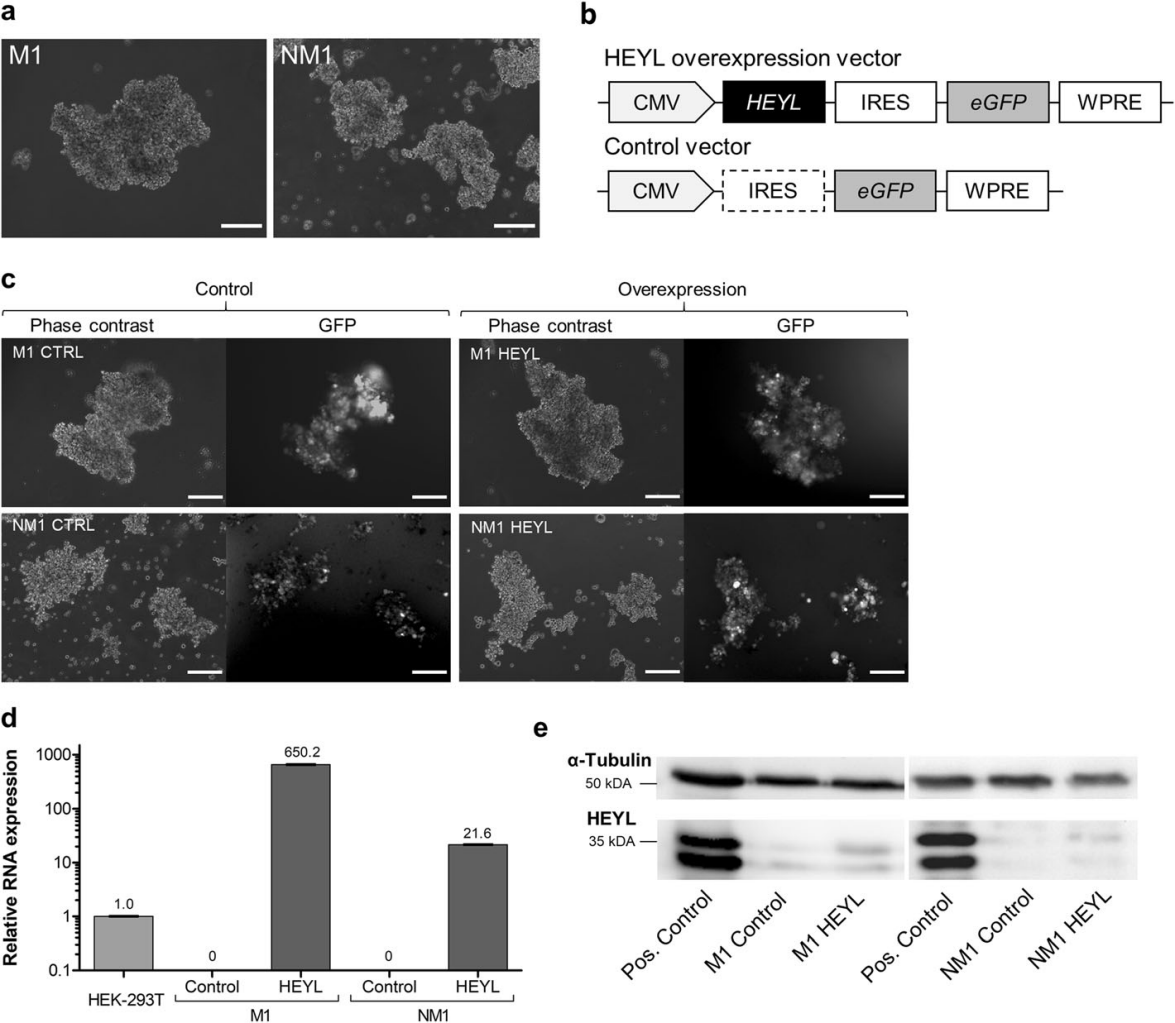
Lentiviral mediated HEYL overexpression in patient-derived colorectal cancer cells. **a** Morphology of patient-derived colorectal cancer spheroid cultures (M1 and NM1). Phase contrast, scale bar: 200 μm. **b** Schematic overview of relevant functional sequences of the lentiviral HEYL overexpression and control vectors. CMV, human cytomegaly virus promotor; IRES, internal ribosome entry site; *eGFP*, enhanced green fluorescent protein; WPRE, woodchuck hepatitis posttranscriptional regulatory element. **c** The lentivirally encoded gene product GFP is expressed in patient-derived CRC spheroid cultures. Phase contrast and GFP fluorescence, scale bar: 200 μm. **d** NM1 and M1 cells lack endogenous *HEYL* mRNA expression. *HEYL* mRNA is detectable in patient-derived CRC spheroid cultures after transduction with the lentiviral HEYL overexpression vector, but not with the control vector. Relative expression levels were quantified via qRT-PCR as fold-expression calibrated to HEK-293 T control with *GAPDH* as internal control. Depicted are mean and standard deviation (*n* = 3). **e** HEYL protein is weakly expressed in patient-derived CRC spheroid cultures with a 1.7-fold and a 2.1-fold higher optical density for the M1 and NM1 *HEYL-*transduced cells compared to control, respectively. Western Blot with α-Tubulin used as internal control; HEYL band at ca. 35 kDa (upper band); HEYL-overexpressing adherent HEK-293 T were used as a positive (pos.) control

To functionally assess the effect of HEYL expression on CRC metastasis formation, we modified the expression of HEYL in primary CRC cells. Therefore, we lentivirally overexpressed HEYL in two patient-derived CRC spheroid cultures, NM1 and M1 (Fig. 2a, Additional file 2), showing distinct metastasis formation patterns after xenotransplantation into immune deficient NSG mice: After sub-renal capsular transplantation, wildtype M1 cells regularly disseminate into the bone marrow and form diffuse tumor infiltration of the neck (nuchal stroma metastasis) as well as distant metastases in the liver and the lungs. In contrast, wildtype NM1 cells regularly show tumor cell dissemination into the bone marrow, but do not metastasize spontaneously.

To perform the overexpression, the human *HEYL* cDNA sequence was cloned into the third-generation self-inactivating lentiviral vector pRRL_CMV_*eGFP*_WPRE (Fig. 2b) and the functionality of the vector was validated in HEK293T cells (Additional file 3+ 4). After transduction, selection and expansion of GFP-positive cells, the majority of transduced cultures was at least 80% positive for GFP, while NM1 control expansion cultures harbored 50–69% of GFP expressing cells (Fig. 2c, Additional file 4). Whereas *HEYL* mRNA expression was undetectable in control vector-transduced CRC cultures, *HEYL* mRNA was strongly overexpressed in *HEYL*-transduced cultures (M1 = 650-fold; NM1 = 22-fold higher compared to HEK-239 T untransduced control) further validating successful lentiviral gene transfer (Fig. 2d). On protein level, both cultures showed a weak HEYL expression. In line with the qRT-PCR results, HEYL protein was 1.7-fold and 2.1-fold higher expressed in M1 and NM1 *HEYL*-transduced cells, respectively, compared to controls (Fig. 2e), indicating overexpression of HEYL protein in patient-derived CRC spheroid cultures.

### Overexpression of HEYL impairs spontaneous metastasis formation in vivo

After sub-renal capsular xenotransplantation into NSG mice, cells have to leave the primary tumor, intravasate into the blood system and proliferate at a distant organ site in order to form metastasis. To address whether HEYL plays a role in spontaneous metastasis formation in vivo, we transplanted either 1 × 10^5^ HEYL overexpression or control vector-transduced M1 and NM1 cells under the kidney capsule of highly immune deficient NSG mice (*n* = 6 per group) (Table 1).

**Table 1:** Characteristics of mice and tumors within the sub-renal capsular xenotransplantation model

Experiment	Characteristics	Control	HEYL	*p*-value
M1	Number of mice (+death)	6	5 (+ 1)	
	Tumor weight [g] median (range)	1.25 (0.75–1.8)	1.3 (0.9–1.43)	0.952
	Time to tumor growth [d] median (range)	75 (71–77)	68 (65–68)	< 0.0001
NM1	Number of mice	5	5	
	Tumor weight [g] median (range)	3.6 (2.8–6.8)	2.9 (1.6–4.64)	0.213
	Time to tumor growth [d] median (range)	91 (77–100)	99 (91–106)	0.101

**Fig. 3:**
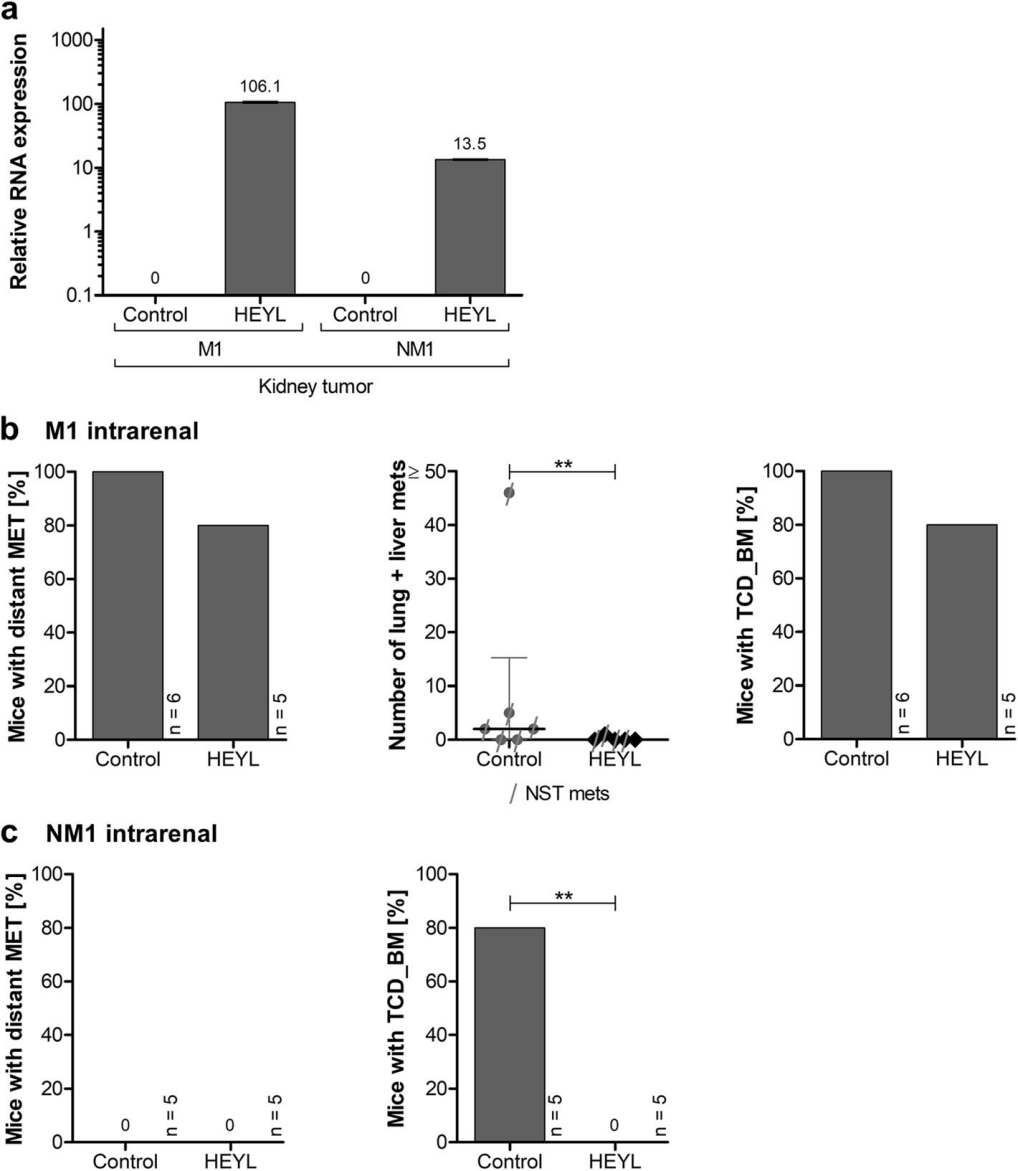
In vivo metastasis formation and tumor cell dissemination of HEYL-overexpressing cells after sub-renal capsular xenotransplantation. **a** *HEYL* mRNA expression in kidney capsule xenograft tumors after transduction of cells with lentiviral overexpression vectors and xenotransplantation into mice. Relative expression levels were quantified via qRT-PCR as fold-expression calibrated to HEK-293 T control with *GAPDH* as internal control. Data for one representative primary tumor is shown per condition. Depicted are mean and standard deviation (n = 3). **b**-**c** Metastasis formation of M1 (**b**) and NM1 (**c**) cells after lentiviral HEYL overexpression compared to control. Shown as a bar chart is the proportion of mice with and without any distant metastases to solid organs (left), as a scatterplot the total number of liver (and lung) metastases with labelling of metastases at other organ sites (middle) and as bar chart the proportion of mice with tumor dissemination into the bone marrow (right). Total numbers of mice (n) within the bar charts are depicted on the right side of the bar. Median and interquartile range are shown within the scatterplot. Significant differences are marked with an asterisk (*, *p* < 0.05; **, *p* < 0.01). MET, metastases; TCD_BM, tumor cell dissemination into the bone marrow; NST, nuchal stroma metastasis

Xenograft tumors formed in all groups, and all tumors originating from HEYL-overexpressing cells stably expressed *HEYL* (Fig. 3a). In M1 cell-transplanted mice, xenograft tumors showed similar tumor weights (median: control 1.25 g, HEYL 1.3 g; *p* = 0.952;), but slightly decreased time to tumor growth in the HEYL group (median: control 75 d, HEYL 68 d; *p* = 0.0001, HEYL group median < 10% of the control group median). In NM1 cell-transplanted mice, no differences in tumor weight or time to tumor growth were detectable (median tumor weight: control 3.6 g, HEYL 2.9 g; *p* = 0.213; median time to tumor growth: control 91 d, HEYL 99 d; *p* = 0.101).

Distant metastases formed in all M1 cell-transplanted mice of the control group (6/6) but only in 80% of the HEYL group (4/5; *p* = 0.251) (Fig. 3b). All metastasized mice presented nuchal stroma metastasis. 66.7% of the control group (4/6), but only 20% (1/5) of the HEYL group also showed metastatic spread to the liver or lungs (*p* = 0.122). Of note, the absolute numbers of liver and lung metastases were reduced following HEYL overexpression from a median of 2 in the control group (interquartile range (IQR): 0–15.25) to a median of 0 in the HEYL group (IQR: 0–0.5; *p* = 0.009). As the bone marrow can constitute a niche for CRC cells with metastasizing capacity [27], we further assessed tumor cell dissemination into the bone marrow as defined by the outgrowth of tumor spheroids from bone marrow cells within 1 month after isolation. While tumor cell dissemination into the bone marrow was detectable in all mice transplanted with control vector-transduced M1 cells (6/6), it was found in only 80% of the mice transplanted with HEYL expressing M1 cells (4/5; *p* = 0.251).

The mice transplanted with control or *HEYL*-transduced NM1 cells did not form distant metastases (Fig. 3c) (control 0/5, HEYL 0/5). However, while 80% of the control group mice (5/6) showed detectable tumor cell dissemination into the bone marrow, tumor cell dissemination was not detected in any of the HEYL group mice (0/6; *p* < 0.0001).

The fact that HEYL overexpression induced significant reduction in the number of metastases in M1 and loss of detectable tumor cell dissemination into the bone marrow in NM1 cell-transplanted mice, indicates that HEYL may act as a negative regulator of metastatic capacity in CRC.

### HEYL overexpression does not impair metastatic capacity downstream of intravasation

**Fig. 4:**
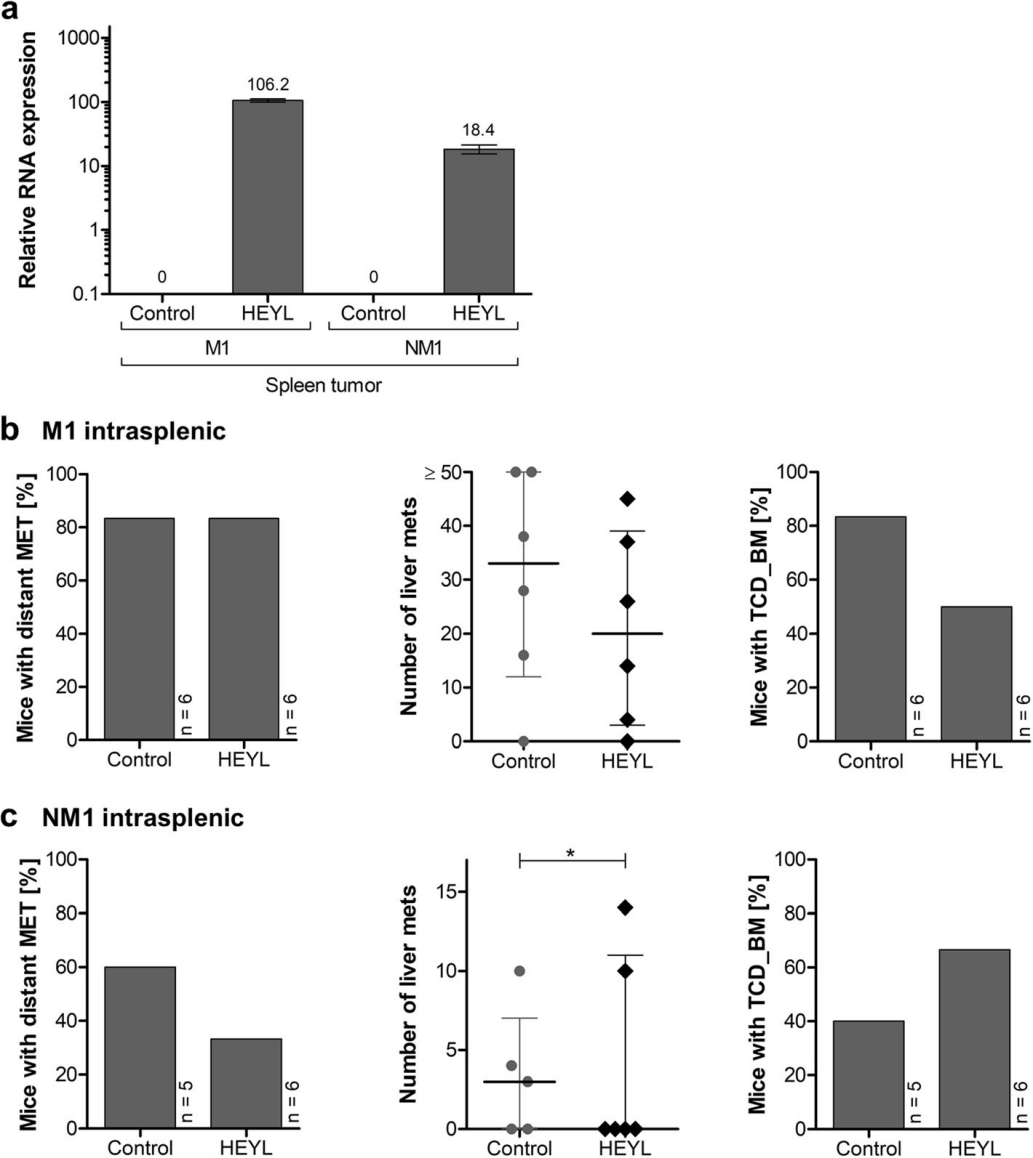
In vivo metastasis formation and tumor cell dissemination of HEYL-overexpressing cells after intrasplenic xenotransplantation. **a** *HEYL* mRNA expression in spleen xenograft tumors after transduction of cells with lentiviral overexpression vectors and xenotransplantation into mice. Relative expression levels were quantified via qRT-PCR as fold-expression calibrated to HEK-293 T control with *GAPDH* as internal control. Data for one representative primary tumor is shown per condition. Depicted are mean and standard deviation (n = 3). **b**-**c** Metastasis formation of M1 (**b**) and NM1 (**c**) cells after lentiviral HEYL overexpression compared to control. Shown as a bar chart is the proportion of mice with and without any distant metastases to solid organs (left), as a scatterplot the total number of liver metastases with labelling of metastases at other organ sites (middle) and as bar chart the proportion of mice with tumor dissemination into the bone marrow (right). Total numbers of mice (n) within the bar charts are depicted on the right side of the bar. Median and interquartile range are shown within the scatterplot. Significant differences are marked with an asterisk (*, p < 0.05; **, p < 0.01). MET, metastases; TCD_BM, tumor cell dissemination into the bone marrow; NST, nuchal stroma metastasis

To narrow down possible steps of the metastatic cascade in which HEYL plays a role, we transplanted M1 and NM1 cells transduced with HEYL or control vector into the spleen of NSG mice (intrasplenic) (*n* = 6 per group) (Fig. 4, Table 2). This way, the cells are directly placed into the circulatory system and thereby skip the potentially rate-limiting steps necessary for intravasation.

**Table 2:** Characteristics of mice and tumors within the intrasplenic xenotransplantation model

Experiment	Characteristics	Control	HEYL	*p*-value
M1	Number of mice	6	6	
	Tumor weight [g] median (range)	1.68 (0.2–2.3)	2.45 (2.1–3.0)	0.022
	Time to tumor growth [d] median (range)	42 (32–44)	36.5 (27–44)	0.447
NM1	Number of mice (+death)	5 (+ 1)	6	
	Tumor weight [g] median (range)	4.97 (0.4–5.8)	2.65 (1.3–4.1)	0.222
	Time to tumor growth [d] median (range)	50 (50–54)	50 (35–54)	0.174

After intrasplenic xenotransplantation, again, xenograft tumors formed in all groups (Fig. 4a). Tumor sizes were slightly increased following xenotransplantation of HEYL expressing M1 cells (median: control 1.68 g, HEYL 2.5 g; *p* = 0.022) without a relevant difference in time to tumor growth (median: control 42 d, HEYL 36.5 d; *p* = 0.447). Tumor weight and time to tumor growth were similar in both NM1 cell-transplanted groups (median tumor weight: control 4.97 g, HEYL 2.65 g; *p* = 0.222; median time to tumor growth: control 50 d, HEYL 50 d; *p* = 0.174).

Following intrasplenic transplantation of M1 cells (Fig. 4b) liver metastases formed in 83% of all mice (control 5/6, HEYL 5/6; *p* = 1.0), other distant metastases were not observed. M1 control group mice harbored a median of 30 liver metastases per mouse (IQR: 12–50), M1 HEYL group mice only a median of 20 liver metastases (IQR: 3–30; *p* = 0.300). Similarly, disseminated tumor cells in the bone marrow were found in 83% of the control group (5/6), but only 50% of the HEYL group mice (3/6; *p* = 0.221).

Intrasplenic xenotransplantation of NM1 cells (Fig. 4c) led to liver metastases formation in 60% of the control group mice (3/5) and 33% of the HEYL group mice (2/6; *p* = 0.377) with a median number of liver metastases of 3 (IQR: 0–7) in the NM1 control group and 0 in the HEYL group (IQR: 0–11). Of note, only two HEYL group mice developed metastases, however, these two mice tended to harbor a higher absolute number of metastases than metastasized mice of the control group (median number of liver metastases: control 4; HEYL 12) leading to an increase in the number of liver metastases in the zero-inflated binomial regression model (*p* = 0.018). Disseminated tumor cells were detected into the bone marrow of 40% of the control group mice (2/5) and 67% of the HEYL group mice (4/6; p = 0.377).

In summary, our data indicate that HEYL overexpression in patient-derived CRC cells impairs their metastasis formation capacity as detected by a decreased number of distant metastases or lack of mice with disseminated tumor cells into the bone marrow following sub-renal capsular xenotransplantation. In contrast to that, unchanged or even slightly increased numbers of liver metastases were found after intrasplenic xenotransplantation where the tumor cells have direct access to the blood circulation. This indicates that HEYL may impede metastatic capacity prior to or required for the intravasation of metastasis-initiating cells but does not impair their metastatic capacity after overcoming this rate limiting step.

## Discussion

The notch pathway has been shown to be involved in the metastatic spread of various tumor entities [8, 9], whereas the impact of its target gene *HEYL* in the context of metastasis so far remains unclear. While our analysis of publicly available CRC datasets suggested an association of high *HEYL* RNA levels with advanced CRC and CRC metastasis, functional analysis in xenotransplantation models of patient-derived cells pointed towards a metastasis-inhibiting role of HEYL with a decrease in the metastasis formation capacity of patient-derived CRC cells in the sub-renal capsular xenotransplantation model after HEYL overexpression.

The discrepancies between the correlation of CRC HEYL mRNA levels with patient metastasis and the functional analysis in xenotransplantation models may be explained by several reasons: An important limitation of mRNA measurements from patient material is that they may not solely resemble mRNA expression in the tumor cells but also mRNA expression in the cells of the tumor microenvironment. Furthermore, it cannot be discriminated between the expression in metastasis-initiating cells and other tumor bulk cells. HEYL expression might also be transient and change during different steps of the metastatic cascade. Besides, mRNA levels do not necessarily correlate with protein levels [34, 35]. The effect of HEYL could also be cell- or tumor-specific. Published studies with experimental modulations of HEY factor expression had little overlap in their gene expression data [12, 36–39]. It has been suggested by Heisig et al. earlier, that HEY factors may rather modulate existing transcription conditions instead of inducing entirely new expression patterns [12].

To illumine the functional impact of HEYL in the context of metastasis, we performed xenotransplantation experiments with patient-derived spheroid cultures overexpressing HEYL. Patient-derived spheroid cultures phenotypically reflect the original patient tumor [27, 40–42], show growth and diffusion characteristics of micrometastases [43] and were shown to enrich for tumor-initiating cells (TIC) [27, 44, 45]. Sub-renal capsular and intrasplenic xenotransplantation of patient-derived spheroid cultures allow the examination of different steps of the metastatic cascade in vivo and showed regular growth of tumors and reproducible patterns of metastases. Of note, current xenotransplantation models do not allow assessing patients’ immune system or the tumor microenvironment as the mice are immunocompromised and stromal tissues are murine. Prospectively, 3D-tissue models might constitute an opportunity to further illuminate these aspects and to further minimize animal experiments in metastasis research [46, 47].

In all intrasplenic xenotransplantation experiments, distant metastases were found solely in the liver. This is the main organ of distant metastases in patients with colorectal cancer as well [48]. After sub-renal capsular xenotransplantation of M1 cells, metastases were also detected in the lungs and the nuchal stroma. We hypothesize that this difference in the sites of metastases is due to the anatomy of the vasculature at the primary transplantation sites. To metastasize, cells capable of giving growth to a new tumor first need to arrive at their new site of growth. This can occur by passive transport of tumor cells into downstream organs [49] as well as active homing of cells into specific organs according to the seed and soil hypothesis by Paget [50, 51]. After sub-renal capsular xenotransplantation, the total number of lung and liver metastases was significantly reduced in the M1 culture. A possible confounder in this experiment might have been a shorter time to tumor growth in the HEYL group. However, the difference in time is quite small (difference = 7 days, less than 10%) and the tumor weight was similar between groups. In mice transplanted with NM1 HEYL-overexpressing cells, particularly the tumor cell dissemination into the bone marrow was inhibited. In contrast to the sub-renal capsular transplantation, intrasplenic xenotransplantation places the cells directly into the blood circulation. The impeding effect of HEYL on metastasis formation in the sub-renal capsular but not in the intrasplenic xenotransplantation models indicates that HEYL inhibits steps of the metastatic process prior to or required for the entrance of metastasis-initiating cells into the vasculature (local migration, invasion or intravasation) [49]. Since we observed different patterns of metastases for the M1 and NM1 cells derived from two different CRC patients, there appears to be considerably interindividual heterogeneity in the effect of HEYL overexpression on metastatic capacity. Importantly, our results from different in vivo models point towards a negative regulatory role of HEYL in CRC metastasis formation by inhibiting intravasation of metastasis-initiating cells.

In different human cancers, the numbers of detectable circulating tumor cells (CTCs) are associated with a worse outcome [52–54]. Interestingly, recent evidence indicates that distinct Notch pathway members are expressed in CTCs and may positively influence their metastatic potential [55–57]. So far the role of HEYL in CTCs remains completely unclear. Our finding of decreased intravasation following HEYL overexpression in our xenotransplantation models suggests that also in CRC patients, higher HEYL expression levels in individual cells may negatively influence their capability to enter the circulation and become a CTC. Coupling single cell expression analyses of circulating tumor cells with functional readouts may be able to address the role of a potential transient HEYL expression in regulating their metastatic capacity of individual circulating tumor cells in CRC patients.

Further studies are needed to understand the mechanism and signaling cascades of the impeding effect of HEYL on metastasis formation and to examine their potential as therapeutic targets against metastatic CRC.

## Conclusion

Metastasis and its consequences are the main causes for death in CRC patients, and rational targeting strategies against metastasized CRC as well as a deepened understanding of the mechanisms enabling metastatic cells to leave the primary site, colonize distant organs and form metastases are urgently needed. Our study reveals an association between high expression of the Notch target gene *HEYL* and metastatic disease in CRC patients. Functional analysis indicates a negative regulatory role for HEYL in spontaneous metastasis formation and suggests that HEYL affects metastatic capacity already before or during the intravasation of metastasis initiating cells. These findings provide the basis for a further mechanistic understanding of metastasis formation in human CRC.

## Supplementary information

**Additional file 1.** Sequences of primers used for qRT-PCR

**Additional file 2.** Characteristics of colorectal cancer patients whose tumor cells were used in this study

**Additional file 3.** Lentiviral HEYL overexpression in HEK-293 T cells

**Additional file 4.** Percentage of GFP-positive cells in the HEYL-overexpressing and control cells

## Data Availability

Clinical data, sample information and corresponding RNA-Seq gene expression data of patients with colon adenocarcinoma (COAD) and rectal adenocarcinoma (READ) from The Cancer Genome Atlas (TCGA) are available via UCSC Xena (http://xena.ucsc.edu). Whole human genome expression DNA microarray data sets of patients with CRC are available via NCBI GEO (https://www.ncbi.nlm.nih.gov/sites/GDSbrowser?acc=GDS4393 and https://www.ncbi.nlm.nih.gov/sites/GDSbrowser?acc=GDS4396). All other data generated during this study are included in this published article and its supplementary information files.

## Abbreviations

AJCC: American Joint Committee on Cancer
COAD: Colon adenocarcinoma
CRC: Colorectal cancer
CTC: Circulating tumor cell
eGFP: Enhanced green fluorescent protein
EMT: Epithelial-mesenchymal transition
FACS: Fluorescent associated cell sorting
GEO: Gene Expression Omnibus
HESR: Hairy and enhancer of split-related
HIV-1: Human immunodeficiency virus type 1
IQR: Interquartile range
IS: Intrasplenic
MET: Metastases
MOI: Multiplicities of infection
MSI: Microsatellite instability
NCBI: National Center for Biotechnology Information
NSG: NOD.Cg-Prkdc^scid^ Il2rg^tm1Wjl^/SzJ
NST: Nuchal stroma metastasis
OS: Overall survival
READ: Rectal adenocarcinoma
SRC: Sub-renal capsular
TCD_BM: Tumor cell dissemination into the bone marrow
TCGA: The Cancer Genome Atlas
TIC: Tumor-initiating cells
