# Supplementary material for: The notch target gene HEYL modulates metastasis forming capacity of colorectal cancer patient-derived spheroid cells in vivo
Source: BMC Cancer. 2019 Dec 3;19:1181. doi: 10.1186/s12885-019-6396-4 (PMC6892194; doi:10.1186/s12885-019-6396-4)
Supplement: Supplementary file 1 — Additional file 1. Sequences of primers used for qRT-PCR [file 12885_2019_6396_MOESM1_ESM.pdf]

Additional file 1. Sequences of primers used for qRT-PCR.

Primer	Sequence (5‘ – 3‘)
***GAPDH* forward**	ACCCAGAAGACTGTGGATGG
***GAPDH* reverse**	TCTAGACGGCAGGTCAGGTC
***HEYL* forward**	GGCTGCTTACGTGGCTGTT
***HEYL* reverse**	GACCCAGGAGTGGTAGAGCAT
