# Supplementary material for: The notch target gene HEYL modulates metastasis forming capacity of colorectal cancer patient-derived spheroid cells in vivo
Source: BMC Cancer. 2019 Dec 3;19:1181. doi: 10.1186/s12885-019-6396-4 (PMC6892194; doi:10.1186/s12885-019-6396-4)
Supplement: Supplementary file 2 — Additional file 2. Characteristics of colorectal cancer patients whose tumor cells were used in this study [file 12885_2019_6396_MOESM2_ESM.pdf]

Additional file 2. Characteristics of colorectal cancer patients whose tumor cells were used in this study.

Primary sphere culture	M1	NM1
**Age-range at surgery [years]**	60-70	60-70
**Sex**	male	male
**UICC classification**	IV	IV
**Localisation of extracted tissue**	Colon transversum	Lung metastasis
