# Supplementary material for: The notch target gene HEYL modulates metastasis forming capacity of colorectal cancer patient-derived spheroid cells in vivo
Source: BMC Cancer. 2019 Dec 3;19:1181. doi: 10.1186/s12885-019-6396-4 (PMC6892194; doi:10.1186/s12885-019-6396-4)
Supplement: Supplementary file 3 — Additional file 3. Lentiviral HEYL overexpression in HEK-293 T cells [file 12885_2019_6396_MOESM3_ESM.pdf]

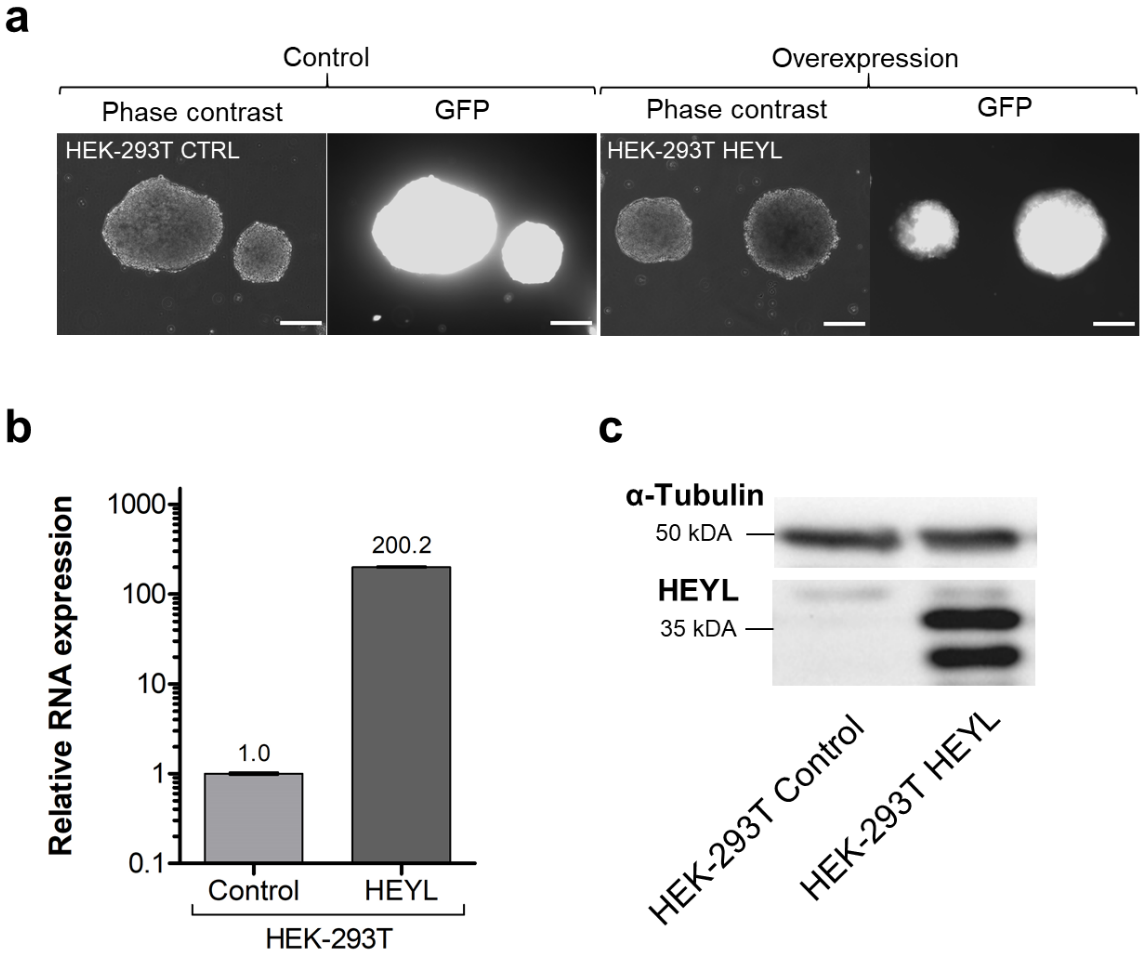

Additional file 3. Lentiviral HEYL overexpression in HEK-293T cells.

(A) Validation of the translation of lentivirally encoded gene products into proteins in HEK-293T spheroid cultures *via* detection of GFP expression. Phase contrast and GFP fluorescence, scale bar: 200µm. (B) HEK-293T cells show a detectable *HEYL* RNA expression with a 200-fold increase in *HEYL*-transduced HEK-293T spheroid cultures. Relative expression level was quantified via qRT-PCR as fold-expression calibrated to HEK-293T control with *GAPDH* as internal control. Depicted are mean and standard deviation (n = 3). (C) HEK-293T control cells only show a very weak HEYL expression. *HEYL*-transduced HEK-293T spheroid cultures strongly overexpress HEYL (98-fold higher optical density) measured *via* Western Blot. α-Tubulin was used as internal control; HEYL-overexpressing adherent HEK-293T were used as a positive control.
