# Supplementary material for: The notch target gene HEYL modulates metastasis forming capacity of colorectal cancer patient-derived spheroid cells in vivo
Source: BMC Cancer. 2019 Dec 3;19:1181. doi: 10.1186/s12885-019-6396-4 (PMC6892194; doi:10.1186/s12885-019-6396-4)
Supplement: Supplementary file 4 — Additional file 4. Percentage of GFP-positive cells in the HEYL-overexpressing and control cells [file 12885_2019_6396_MOESM4_ESM.pdf]

Affiliation file 4. Percentage of GFP-positive cells in the HEYL-overexpressing and control cells.

**Culture**	**GFP-positive cells before *in vitro/ in vivo* experiments**
**HEK-293T control**	96-99 %
**HEK-293T HEYL**	75-98 %
**M1 control**	84 % (SRC, IS)
**M1 HEYL**	82 % (SRC, IS)
**NM1 control**	50 % (SRC), 69 % (IS)
**NM1 HEYL**	80 % (SRC), 86 % (IS)

Measured *via* flow cytometry after selection of GFP-positive cells *via* FACS and expansion of the cells. SRC, sub-renal capsular; IS, intrasplenic.
